# Supplementary material for: Fully automated image-based estimation of postural point-features in children with cerebral palsy using deep learning
Source: R Soc Open Sci. 2019 Nov 6;6(11):191011. doi: 10.1098/rsos.191011 (PMC6894590; doi:10.1098/rsos.191011)
Supplement: Supplementary material [file rsos191011supp1.docx]

^[[1]](#footnote-1)^

Supplementary Document

Ryan Cunningham, *Member, IEEE*, María B. Sánchez, Penelope B. Butler, Matthew J. Southgate, and Ian D. Loram, Member, IEEE

| 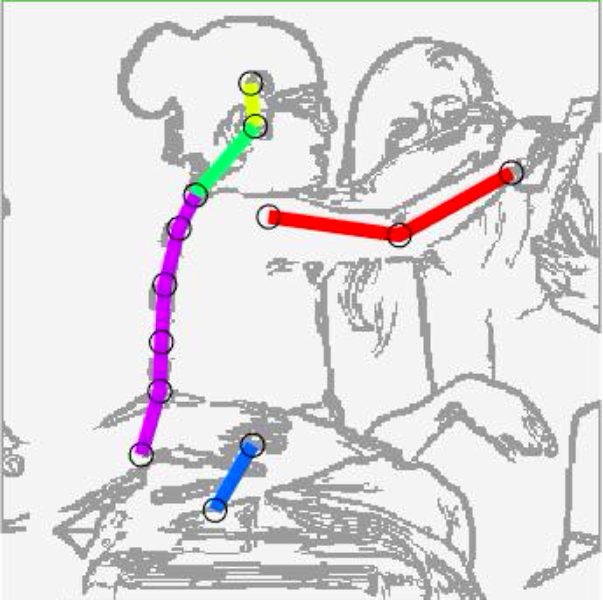 |
| --- |
| **Figure 1. Supplementary videos.** With this manuscript we have published a set of videos showing the entire data set in video form (represented as cartoon images*). Each video is a concatenation of multiple sessions and trials for an individual child, representing a unique test batch for each of the 12 neural networks. The filename indicates the batch number and the test set, where ‘testing’ represents test batch 1, and ‘validation’ represents test batch 2 (see section *II. Methods*, subsection *G. Training and Cross-Validation* in the full article). The graphic (above) shows an example still image taken from one of the videos. The child is represented by a cartoon outline (generated automatically using canny edge detection in Matlab), with the neural network output superimposed as coloured lines and black circles; the coloured lines represent the 10 individual segments (see figure 1 in the full article), and the circles represent the 13 point-features.  *the actual colour images cannot be shown for ethical reasons, therefore cartoon images are shown to illustrate. |

| Max-Pooling  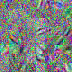 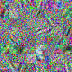 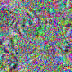 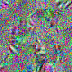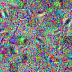 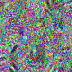 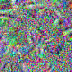 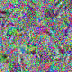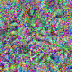 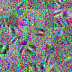 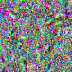 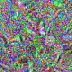 | Mean-Pooling  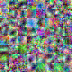 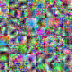 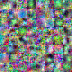 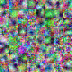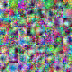 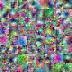 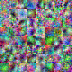 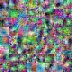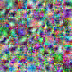 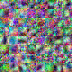 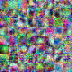 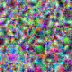 |
| --- | --- |
| **Figure 2. Neural network filters.** For each of the 12 max-pooling and 12 mean-pooling neural networks we give the first layer of filters for visual comparison. Each of the 12 tiles shows 64 filters arranged in 8x8 blocks. Note the distinct difference in max-pooling and mean-pooling filters. The most distinctive feature of the max-pooling filters is the tendency to model oriented strokes or edge-like features. Whereas the mean-pooling filters model convex-hull and bulbus shape pattern, with some quasi-edge features. | |

| 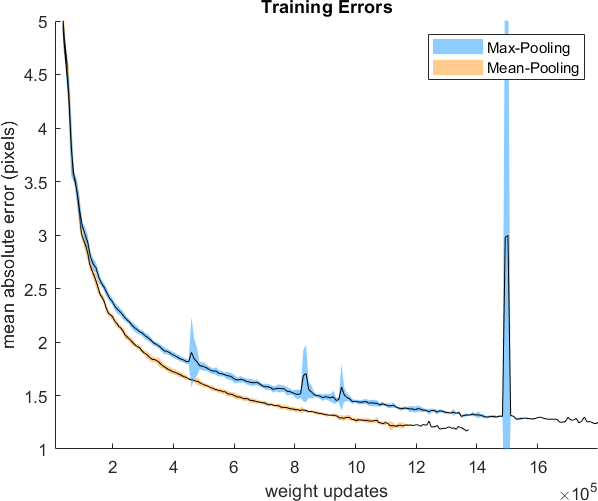 | 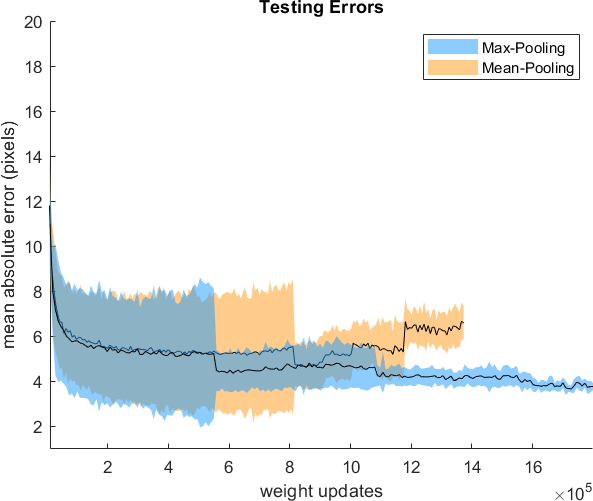 |
| --- | --- |
| **Figure 3. Neural network errors during learning.** The learning curves are given for both max-pooling and mean-pooling neural networks. Errors have been interpolated and, in each graphic, the solid black line represents the mean over all 12 participants, and the solid colour patch represents the standard deviation over all 12 participants. Observation 1: The training error converges more rapidly and to a lower point in the mean-pooling network. Observation 2: There is little to separate the testing errors; the confusing rise in errors after 500k weight updates arises from early stopping of some of the networks, which results in the illusion of apparent rise in error. Therefore, the representative testing error curve is in the first 500k weight updates. Observation 3: The max-pooling network trained for slightly longer than the mean-pooling network. Observation 4: The max-pooling network tended to experience brief moments of high error gradient during training. | |

| 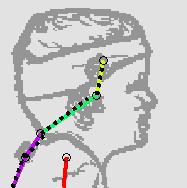  **Neck: 2.4° Head: 3.3°**  **‘Aligned’** | 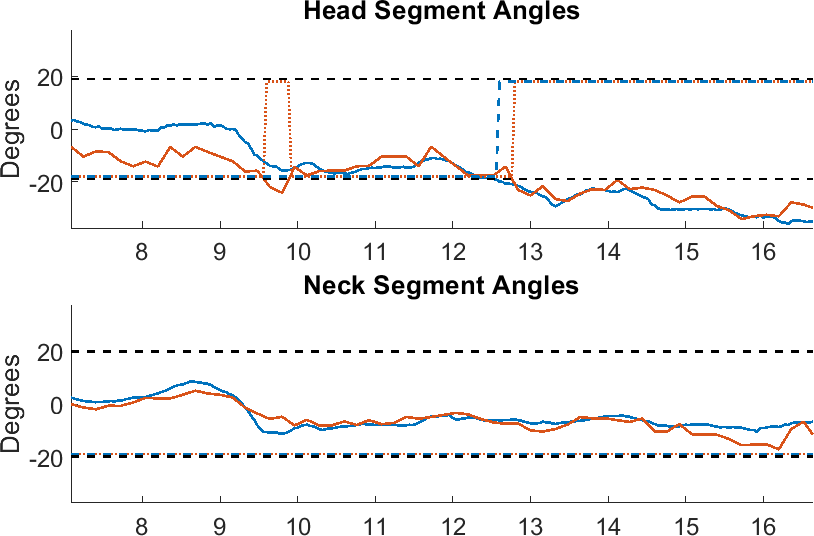 | 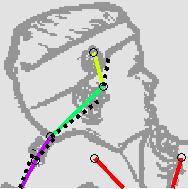  **Neck: -6.7° Head: -26.7°**  **Head Extension** |
| --- | --- | --- |
| 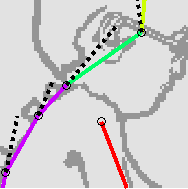  **UT: 20.4° MT: 17.8°**  **UT-MT Flexion** | 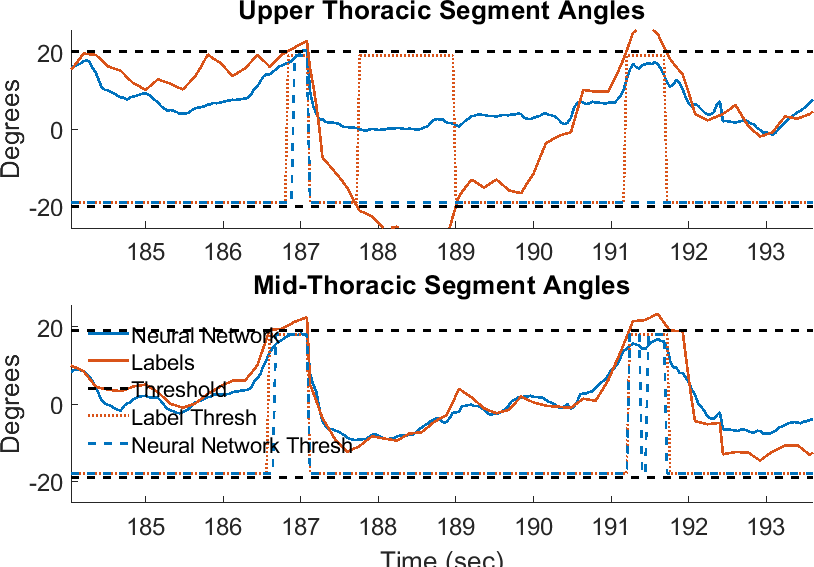 | 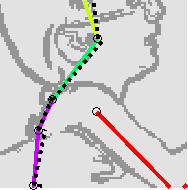  **UT: 2.2° MT: -6.6°**  **‘Aligned’** |
| **Figure 4. Neural network classification of segment alignment*.** The 4 graphics on the outer left/right panels each show an image** of a child, zoomed around the point of interest, with the neural network-predicted point-features (circles). Point-features are connected by colour solid lines which denote head (yellow), neck (green), trunk (pink), and arm (red). The dashed black line shows the reference angle for each segment (the ‘aligned’ segment angle). The numbers in degrees below each of the 4 outer graphics note the deviation of the coloured line from the dashed line (difference from ‘alignment’). The timeseries panel in the middle shows, for the relevant segments, the deviation from the aligned angle, threshold lines, and threshold versions of the segment angles, for both labels, and neural network, over a video sequence. The black arrows going from graphic to the timeseries shows the approximate point in time from which the graphics and point-feature predictions were extracted. The thresholds used in this analysis were taken from table 2 (Optimized Thresholds). | | |
| *our definition of aligned posture is the mean over all sessions and trials for a given child  **the actual colour images used in the analysis cannot be shown for ethical reasons, therefore cartoon images are shown to illustrate. | | |
|  | | |

| 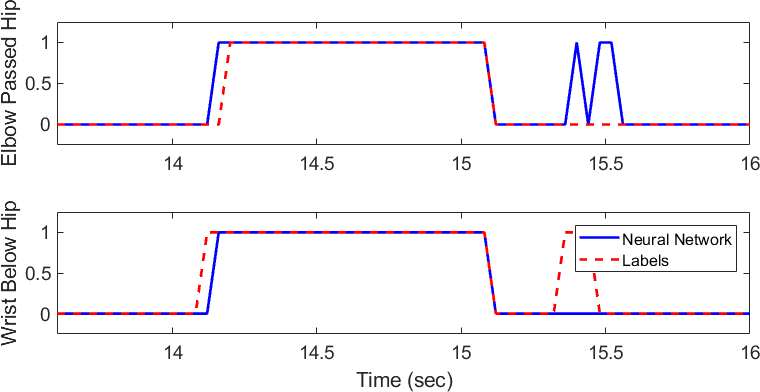 | | |
| --- | --- | --- |
| 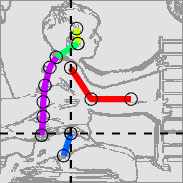 | 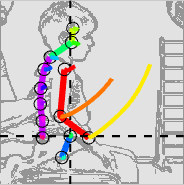 | 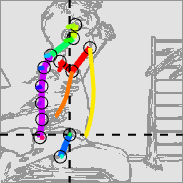 |
| **Figure 5. Representative results of neural network classification of external support via the arm.** Top panel shows timeseries of neural network (blue) and labels (red) classification of arm in contact with the body for both the elbow (top) and wrist (bottom) point-features. Bottom panel shows snapshots in time of the video sequence (where the black arrows point approximately to the point in time fom which the snapshot was taken). The snapshot comprises an image* of child, the neural network prediction of point-features (circles) and segments (coloured lines joining point-features), a history of previous 15 frames (0.6 seconds) of point-feature motion (colour line traces leading to point-features), and finally, two dashed black lines which represent the lateral and vertical thresholds defined by labels using the upper pelvis marker. The image on the left shows child with arms out in front, and the timeseries correctly shows that the arms are not in contact with the body. The middle image shows a downward motion (colour point-feature line traces), crossing both thresholds, and the timeseries correctly classifying the arms as in contact with the body. The final image shows an upward motion of the arms, and the timeseries correctly shows that the arms are no longer in contact with the body. Some representative amount of misclassification can be seen towards the end of the timeseries, but overall precision is high.  *the actual colour images used in the analysis cannot be shown for ethical reasons, therefore cartoon images are shown to illustrate. | | |

1. [↑](#footnote-ref-1)
